# Cerebellar functional disruption and compensation in mesial temporal lobe epilepsy

**DOI:** 10.3389/fneur.2023.1062149

**Published:** 2023-02-02

**Authors:** Yiqian Peng, Kangrun Wang, Chaorong Liu, Langzi Tan, Min Zhang, Jialinzi He, Yuwei Dai, Ge Wang, Xianghe Liu, Bo Xiao, Fangfang Xie, Lili Long

**Affiliations:** ^1^Department of Radiology, The First Affiliated Hospital of Wenzhou Medical University, Wenzhou, China; ^2^Department of Neurology, Xiangya Hospital, Central South University, Changsha, China; ^3^Department of Neurosurgery, Xiangya Hospital, Central South University, Changsha, China; ^4^Department of Radiology, Xiangya Hospital, Central South University, Changsha, China; ^5^Clinical Research Center for Epileptic Disease of Hunan Province, Xiangya Hospital, Central South University, Changsha, China; ^6^National Clinical Research Center for Geriatric Disorders, Xiangya Hospital, Central South University, Changsha, China

**Keywords:** temporal lobe epilepsy, cerebellum, fMRI, verbal fluency task, independent component analysis (ICA), graph theory

## Abstract

**Background:**

Cerebellar functional alterations are common in patients with mesial temporal lobe epilepsy (MTLE), which contribute to cognitive decline. This study aimed to deepen our knowledge of cerebellar functional alterations in patients with MTLE.

**Methods:**

In this study, participants were recruited from an ongoing prospective cohort of 13 patients with left TLE (LTLE), 17 patients with right TLE (RTLE), and 30 healthy controls (HCs). Functional magnetic resonance imaging data were collected during a Chinese verbal fluency task. Group independent component (IC) analysis (group ICA) was applied to segment the cerebellum into six functionally separated networks. Functional connectivity was compared among cerebellar networks, cerebellar activation maps, and the centrality parameters of cerebellar regions. For cerebellar functional profiles with significant differences, we calculated their correlation with clinical features and neuropsychological scores.

**Result:**

Compared to HCs and patients with LTLE, patients with RTLE had higher cerebellar functional connectivity between the default mode network (DMN) and the oculomotor network and lower cerebellar functional connectivity from the frontoparietal network (FPN) to the dorsal attention network (DAN) (*p* < 0.05, false discovery rate- (FDR-) corrected). Cerebellar degree centrality (DC) of the right lobule III was significantly higher in patients with LTLE compared to HC and patients with RTLE (*p* < 0.05, FDR-corrected). Higher cerebellar functional connectivity between the DMN and the oculomotor network, as well as lower cerebellar degree centrality of the right lobule III, was correlated with worse information test performance.

**Conclusion:**

Cerebellar functional profiles were altered in MTLE and correlated with long-term memory in patients.

## 1. Introduction

Cerebellar alterations are common in mesial temporal lobe epilepsy (MTLE), one of the most prevalent forms of focal epilepsy in adults. The cerebellum is a potential target for seizure control in patients with drug-resistant MTLE because it contributes to cognitive deficiency in MTLE (1–4). Previous studies focused primarily on cerebellar structural abnormalities in MTLE. Cerebellar volume decreased by 4–6.6% in patients with chronic MTLE (5), but not in those with newly diagnosed temporal lobe epilepsy (TLE) (6). Cerebellar volume decreased more with longer disease duration (6–9), higher seizure frequency (6, 10), and a higher total seizure burden (5, 6, 11). Three independent studies reported cerebellar hyperfusion during temporal lobe seizures (12–14). In addition, the cerebellar damage in patients with epilepsy was similar to that in patients with cerebral hypoxia (11). Therefore, cerebellar structural damage was considered to be an acquired abnormality caused by hypoxia, seizure discharges, and hyperfusion during uncontrolled seizures.

The cerebellum is essential for language and long-term memory retrieval, except for motor control (15). Activation in the cerebellum rose with increasing memory load (16, 17). Meanwhile, sequence processing, one of the language functions of the cerebellum, affects the word retrieval strategy during verbal fluency tasks (18–20). Phonemic and semantic verbal fluency tasks are important scales clinically applied for routine and presurgical evaluation of TLE to predict prognosis and postsurgical language outcomes (21–23). According to lesion studies, phonemic verbal fluency was largely attributed to the left frontal cortex and anterior temporal lobe, and semantic verbal fluency was correlated with the left posterior temporal cortex (24). Phonemic verbal fluency requires category switching, causes greater cognitive load, and is, therefore, more dependent on cerebellar function (19, 20). Same as the cerebrum, language function in the cerebellum is lateralized. In right-handed participants, the right posterolateral cerebellum, which is functionally connected to the left prefrontal cortex, supports phonemic processing and linguistic prediction (25, 26). During a phonemic verbal fluency task, the left cerebellum was activated in left-handed participants (25).

Previous studies detected a deviation in cerebellar function in patients with TLE using voxel- and seed-based approaches. During the attentional network test, activation in the cerebellum was reduced in patients with MTLE compared to healthy controls (HC) (27). Impaired functional connectivity between the right dentate nuclei and the left cerebral hemisphere was related to cognitive impairment in MTLE (2, 3). Graph theory analyses provided additional knowledge regarding the role of cerebellar regions in TLE. Centrality statistics represent the importance of a given node in the entire network. In MTLE, cerebellar nodes with higher functional centrality were reported, indicating an attempted compensatory process (28). While the abovementioned approaches unfold cerebellar function at the regional level, the cerebellum is organized as a functional network (29, 30). Group independent component (IC) analysis (group ICA) provides an ideal and robust approach for separating the cerebellum into functionally segregated networks. Phonemic verbal fluency tasks effectively mobilized the cerebellum and provided an ideal tool for studying cerebellar malfunction at the network level. In addition, the clinical application of the Chinese version of the phonemic verbal fluency tasks was delayed due to the linguistic difference between Chinese and Indo-European languages. The involvement of the cerebellum in Chinese verbal fluency remained unclear. Further studies on cerebellar abnormalities of functional networks would deepen our understanding of the pathogenesis of cognitive decline in MTLE.

Based on the Chinese character version of a verbal fluency task, a cross-sectional study aimed to investigate alterations in functional connectivity between cerebellar networks parcellated using group ICA. A comprehensive view of cerebellar functional alterations in MTLE in China was provided using voxel- and seed-based approaches.

## 2. Methods

### 2.1. Participants

A total of 30 consecutive participants with temporal lobe epilepsy and hippocampal sclerosis (HS) were selected from an ongoing prospective cohort (31, 32). All participants visited the outpatient department of Xiangya Hospital between 9 November 2018 and 9 January 2021. A total of 30 HC matched for sex, age, and educational level participated in this study. Sex, age, years of education, age of onset, disease duration, number of antiseizure medications (ASMs), and seizure frequency were extracted from the database. 3DT1, T2WI, and T2WI fluid-attenuated inversion recovery sequences were applied to all participants to identify HS and potential lesions. The diagnosis of HS follows a robust protocol (33): (1) Neuroimagers diagnose HS based on visible changes, including decreased hippocampal volume, increased temporal horn volume, gray–white matter boundary blurring, asymmetric hippocampus, loss of internal structure, and increased T2 signals (34); (2) Reduced hippocampal volume calculated with the online automatic segmentation tool Hipposeg (35); and (3) A disagreement between procedures one and two may be reconciled with a blind rater.

Mesial temporal lobe epilepsy was diagnosed and lateralized based on a comprehensive evaluation of semiology, clinical history, electroencephalography, and magnetic resonance imaging (MRI). All participants in our cohort are right-handed (31, 36). Exclusion criteria included: (1) Those who had a psychiatric or neurological disorder other than MTLE, (2) Those who had a cerebral or cerebellar lesion other than HS, (3) Those who were below 16 or over 65 years of age, (4) Those who were unable to endure or comprehend the procedure, (5) Those who had poor imaging quality, including excessive head motion or poor task effect, and (6) Those who received phenytoin treatment (37).

This study was approved by the Ethics Committee of the Xiangya Hospital of Central South University. Written informed consent was obtained from all participants.

### 2.2. Neuropsychological tests

All participants underwent (1) Montreal Cognitive Assessment (MoCA) (38) to test the overall cognitive function, (2) the Information subtest of the Chinese version of the Wechsler Adult Intelligence Scale (WAIS) as an evaluation for long-term memory (32), (3) Digit Span subsets of WAIS-RC (revised by China) for working memory (39), (4) the Digit Symbol Substitution subset of WAIS-RC to exam the processing speed (32), (5) the Block Design subset of WAIS-RC for perceptual organization, and (6) Verbal Fluency-Chinese Character, Verbal Fluency-Chinese Pinyin (31), and the Boston Naming Test (40) to test the language function.

### 2.3. MRI data acquisition and preprocessing

Magnetic resonance imaging data were collected at the MRI center of the Xiangya Hospital using a Siemens MAGNETOM Prisma 3.0T MR scanner and standard head coils. Structural images were collected using magnetization-prepared rapid acquisition and a gradient echo sequence (field of view, 233 mm; repetition time, 1 s; echo time, 37 ms; flip angle, 9°; 320 × 320 matrix). When participants performed a Chinese character verbal fluency task (31, 41), functional images were obtained with a gradient echo-planar T2-weighted sequence (field of view, 225 mm; repetition time, 1 s; echo time, 37 ms; flip angle, 52°; 90 × 90 matrix) when conducting the Chinese character version of a phonemic verbal fluency task (41).

The task was divided into five blocks, each containing a 30-s rest module and a 30-s task module. In the rest module, a crosshair fixation would be projected on a white background. Participants were instructed to rest while looking at the screen. In each task module, one Chinese character would be displayed on a white background. Participants were instructed to covertly generate words beginning with the given Chinese character.

Raw images were realigned, co-registered, segmented, normalized, and spatially smoothed (6 mm) with the default preprocessing pipeline (31) of Statistical Parametric Mapping 12 (https://www.fil.ion.ucl.ac.uk/spm/). Next, image data were processed using Toolbox CONN v.20.b (42) (http://www.nitrc.org/projects/conn) for further denoising. Head motion, outlying scan detection using an embedded functional outlier, the effect of modules, and signals in the white matter and cerebrospinal fluid were removed as confounders. A bandpass filter [0.009–0.10 Hz] was applied to remove the noise.

### 2.4. Group ICA and network connectivity analysis

Toolbox CONN v.20.b was used for group ICA and subsequent network connectivity analysis. According to a previous study, the cerebellum was segmented into six ICs at the group level (43). Then, group cerebellum ICs were reconstructed back to individual ICs. For each participant, the average blood oxygen level-dependent signal time series in each individual IC was extracted for network connectivity analysis. A generalized linear model (GLM) based on semi-partial correlation was applied to calculate individual-level functional connectivity (42). Individual connectivity values were processed using Fisher transformation (inverse hyperbolic tangent functions) and then compared between HC, left TLE (LTLE), and right TLE (RTLE) using analysis of covariance (ANCOVA) and *post hoc* pairwise comparisons, with sex, age, years of education, and MoCA as covariates of no interest. The significant threshold for network connectivity was *p* < 0.05, false discovery rate- (FDR-) corrected.

### 2.5. Voxel-based analysis

SPM 12 was used to perform a two-level voxel-based analysis of cerebellar activation maps. At the individual level, a task-dependent contrast map was calculated for each participant. At the second level, contrast maps were compared between groups *via* ANCOVA and *post- hoc* pairwise comparison, with sex, age, years of education, and MoCA as covariates of no interest. Clusters were considered significant at *p* < 0.05, FDR-corrected with an additional threshold of 10-voxel minimum cluster size.

### 2.6. Seed-based analysis

The whole brain was parcellated into 210 cortical regions (44), 36 subcortical regions (44), and 26 cerebellar regions (45). For each participant, a GLM based on a bivariate correlation was used to construct a 272 × 272 weighted matrix, which was then transformed into 36 binary matrices with connection density ranging from 5 to 40%, in steps of 1% (46). Under each density, betweenness centrality (BC) and degree centrality (DC) for 26 cerebellar regions were calculated. BC is the frequency that a given node is on the shortest path between all node pairs. DC is the number of suprathreshold connections linked to a particular node. Centralities were compared among the three groups by (1) area under the curve (AUC) across all densities and (2) a subsequent comparison at each density. AUC was calculated using R studio.

The bilateral cerebellar lobule and vermis were defined as seeds in a seed-to-voxel analysis. Functional connectivity between the three regions and whole-brain voxels was computed with CONN v.20.b, based on a GLM and semi-partial correlation. Seed-based functional connectivity was compared among the three groups with ANCOVA and *post-hoc* pairwise comparison, with sex, age, years of education, and MoCA as covariates. Clusters were considered significant at *p* < 0.05, FDR-corrected with an additional minimum cluster size threshold of 10 voxels.

### 2.7. Statistical analysis

IBM SPSS Statistics 23 (https://www.ibm.com/products/spss-statistics) was used for statistical analysis. The distribution of qualitative variables was assessed using the Shapiro–Wilko test. Variables without a normal distribution or homogeneity of variance were compared between the groups with nonparametric approaches and reported as median and interquartile ranges. Sex, age, years of education, and MoCA were compared among the three groups using the chi-square test or the Kruskal–Wallis *H*-test. ANCOVA or Quade nonparametric ANCOVA and *post- hoc* pairwise comparisons were used to compare neuropsychological test scores among the three groups, with sex, age, years of education, and MoCA controlled for confounders. Qualitative and categorical variables were compared between the two patient groups using the two-tailed two-sample *t*-test and Fisher's exact test, respectively.

For functional network connectivity and centrality metrics with a significant group difference, we calculated their partial correlation with age of onset, disease duration, seizure frequency, disease burden = disease duration^*^seizure frequency, number of ASMs, and neuropsychological scores, adjusting for sex, age, years of education, and MoCA.

A *p*-value of < 0.05, FDR-corrected, was considered significant.

## 3. Results

### 3.1. Demographic and clinical data

The three groups did not differ in age, sex, years of education, and MoCA scores. HC outperformed both patient groups in verbal fluency Pinyin (VFP) scores (*p* = 0.004 and 0.02, FDR-corrected for HC vs. LTLE and HC vs. RTLE, respectively). Patients with LTLE also had worse Boston Naming (BN) scores than HC and patients with RTLE (*p* = 0.004 and 0.03, FDR-corrected for LTLE vs. HC and LTLE vs. RTLE, respectively).

Patients with LTLE and RTLE did not differ in their clinical features (see the details in Table 1).

**Table 1:** Participant demographic and clinical data.

	**HC**	**LTLE**	**RTLE**	**Statistic**	**q**
N	30	13	17	-	-
Age, y, median (IQR)	26.0 (18.0)	30.0 (10.0)	26.0 (10.0)	2.31^a^	0.60
Sex, Male/Female	14/16	6/7	8/9	0.00^b^	1.00
Education, median (IQR)	12.3 (3.6)	11.4 (3.6)	11.6 (3.4)	0.98^a^	0.78
MoCA, median (IQR)	27.4 (4.2)	26.7 (2.6)	24.0 (5.5)	9.00^a^	0.07
Information, mean (SD)	15.8 (6.5)	14.5 (6.4)	11.9 (5.3)	1.62^c^	0.49
DSF, median (IQR)	8.2 (1.6)	7.6 (1.1)	7.2 (1.3)	1.92^d^	0.49
DSB, median (IQR)	5.1 (1.8)	4.8 (0.7)	4.9 (1.8)	0.31^d^	0.82
DSST, median (IQR)	60.0 (24.0)	55.0 (18.0)	60.0 (16.0)	0.71^d^	0.68
Block design, mean (SD)	37.6 (8.4)	31.7 (6.1)	34.1 (10.5)	2.21^c^	0.46
VFC, median (IQR)	25.6 (14.7)	16.9 (7.0)	20.4 (5.4)	3.47^d^	0.18
VFP, median (IQR)	47.3 (18.1)	32.9 (11.7)	38.4 (10.7)	7.35^d^	0.03
BN, median (IQR)	27.0 (5.0)	21.0 (10.0)	25.0 (3.0)	5.81^d^	0.05
AOO, y, mean (SD)		20.0 (8.4)	16.2 (6.8)	0.37^e^	0.49
duration, y, mean (SD)		11.5 (8.8)	10.4 (8.4)	1.39^e^	0.82
Disease burden, mean (SD)		22.1 (18.4)	22.5 (20.7)	−0.05^e^	1.00
Febrile convulsion		2 (15.4%)	7 (41.2%)	-^f^	0.64
SGS history		1 (7%)	0 (0%)	-^f^	0.49
Number of ASM				-^f^	0.60
1		8	8		
2		4	9		
3		1	0		
Seizure frequency				-^f^	0.60
Every year		5	2		
Every month		4	9		
Every week		2	4		
Every day		2	2		

a H-value of the Kruskal–Wallis H-test.

b χ^2^ value of the chi-squared test.

c F-value of analysis of covariates.

d F-value of Quade nonparametric analysis of covariates.

e t-value of the two-tailed two-sample t-test.

f Fisher's exact test.

AOO, age of onset; ASM, antiseizure medication; BN, Boston Naming Test; DSB, Digit Span-Backward; DSF, Digit Span-Forward; DSST, Digit Symbol Substitution Test; HC, healthy controls; IQR, interquartile range; LTLE, left temporal lobe epilepsy; MoCA, Montreal Cognitive Assessment; RTLE, right temporal lobe epilepsy; SD, standard deviation; SGS, secondary generalized seizures; VFC, verbal fluency character test; VFP, verbal fluency Pinyin test.

### 3.2. Group-ICA and network connectivity analysis

**Figure 1:**
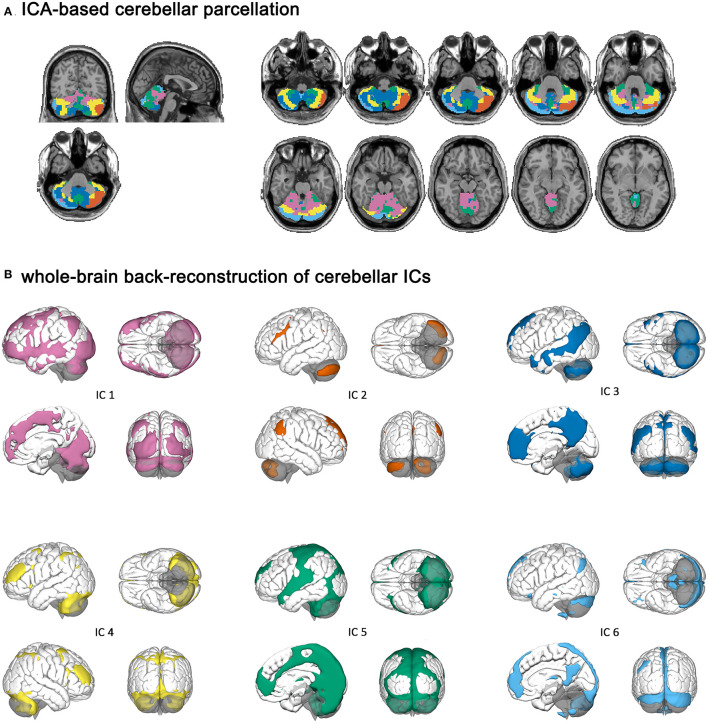
Cerebellar independent components and their cerebral parcellations. **(A)** ICA-based cerebellar parcellation. **(B)** Whole-brain back-reconstruction of cerebellar ICs.

Cerebellar ICs are shown in Figure 1. Cerebellar IC 1 contained bilateral crus I and II, the lobules III–VI, and the vermis III–V. IC 2 and IC 6 had relatively symmetric regions because they included the left crus I and II and the right crus I and II, respectively. IC 6 also contained the left crus I, the bilateral lobules VII and VIII. IC 3 comprised the bilateral crus I and II, the lobules IX, and the vermis IX–X. IC 4 encompassed bilateral crus I and II, and the lobules VI–VIII. IC 5 consisted of the vermis IV–IX and the bilateral lobules VIII and IX.

Independent component (IC) 1 had a cerebral parcellation, which contained the visual region and frontoparietal network (FPN). IC 2 and IC 6 were connected to the left and right FPN, respectively. IC 6 had a cerebral parcellation, which contained regions similar to the default mode network (DMN). IC 3 had connectivity to the DMN, and IC 4 was connected to the dorsal attention network (DAN). IC 5 had a cerebral parcellation consisting of auditory and sensorimotor regions.

**Figure 2:**
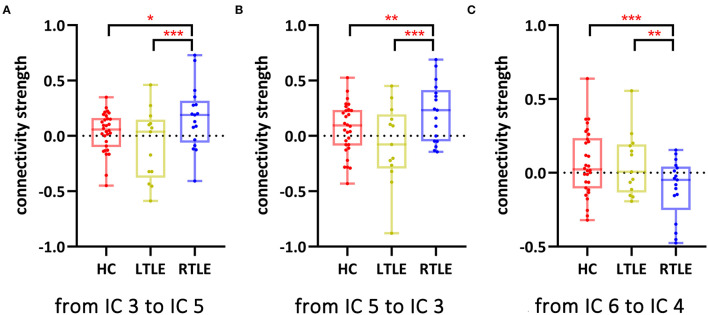
Network functional connectivity with a significant group difference among the three groups. *p < 0.05, FDR-corrected; **p < 0.005, FDR-corrected; ***p < 0.001, FDR-corrected. **(A)** Network functional connectivity from IC 3 to IC 5; **(B)** Network functional connectivity from IC 5 to IC 3; and **(C)** Network functional connectivity from IC 6 to IC 4. HC, healthy controls; IC, independent component; LTLE, left temporal lobe epilepsy; RTLE, right temporal lobe epilepsy.

Functional network connectivity from IC 3 to IC 5 (*p* = 0.04, FDR-corrected), IC 5 to IC 3 (*p* = 0.04, FDR-corrected), and IC 6 to IC4 (*p* = 0.04, FDR-corrected) differed significantly among the three groups. *Post-hoc* comparisons showed that connectivity between IC 3 and IC 5 (Figures 2A, B) was enhanced and connectivity from IC 6 to IC 4 (Figure 2C) was impaired in patients with RTLE compared to HC and patients with LTLE.

### 3.3. Voxel-based analysis

Cerebellar activation maps did not differ among the three groups.

### 3.4. Seed-based analysis

**Figure 3:**
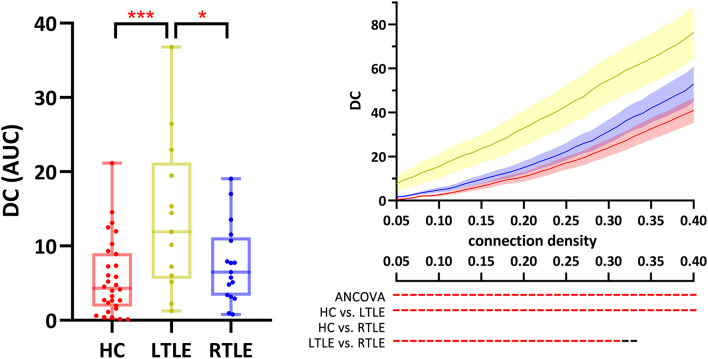
DC of the right cerebellum lobule III was different across the three groups. Red ***, *p* < 0.001, FDR-corrected; Red - & *, p < 0.05, FDR-corrected; black -, *p* < 0.05, uncorrected. The boxplot presents the group comparison of AUC, and the line chart shows the group comparison across all connectivity densities. ANCOVA, analysis of covariates; AUC, area under the curve; DC, degree centrality; HC, healthy controls; IC, independent component; LTLE, left temporal lobe epilepsy; RTLE, right temporal lobe epilepsy.

On the overall scale (Figure 3, boxplot), DC of the right lobule III differed significantly among the three groups (*p* = 0.05, FDR-corrected), as it was elevated in LTLE compared to HCs (*p* = 0.002, FDR-corrected) and RLTE (*p* = 0.03, FDR-corrected). DC of the right lobule III was higher in LTLE than in HC at all densities (*p* < 0.05, FDR-corrected). When the connectivity density was <32%, LTLE also had a higher DC in the right lobule III than RTLE (*p* < 0.05, FDR-corrected; Figure 3, line chart). There was a trend (*p* < 0.05, FDR uncorrected) for the group difference of DC of bilateral lobule X, vermis X, and the BC of the right lobule III, the right lobule X, and vermis X.

Regarding seed-based functional connectivity, we did not observe any significant differences among the three groups.

### 3.5. Correlation analysis

**Figure 4:**
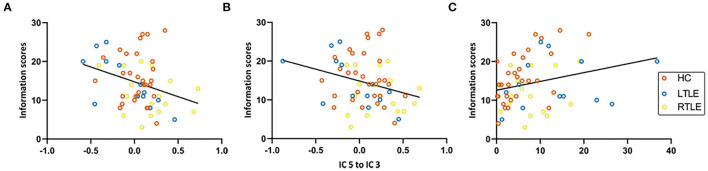
Correlation analysis. HC, healthy controls; IC, independent component; LTLE, left temporal lobe epilepsy; RTLE, right temporal lobe epilepsy. **(A)** Functional connectivity from IC 3 to IC5. **(B)** Functional connectivity from IC 5 to IC 3. **(C)** Degree centrality of right lobule III.

In all participants, weaker connectivity from IC 3 to IC 5 (*r* = −0.33, *p* = 0.02), from IC 5 to IC 3 (*r* = −0.29, *p* = 0.04), and from higher DC of the right lobule III (*r* = 0.31, *p* = 0.02) was related to higher scores on Information Tests (Figure 4).

### 3.6. Sensitivity analysis

One patient with RTLE (female, 29 years old) received topiramate (25 mg, bid). Considering that topiramate was associated with language and cognitive network dysfunction (47, 48), we conducted a sensitivity analysis excluding this patient. The exclusion of this patient did not affect the overall results. In patients with RTLE, functional connectivity between IC 3 and IC 5 and from IC 6 to IC 4 was altered compared to HCs and patients with LTLE. DC of the right lobule III was increased in LTLE compared to HCs (*p* = 0.002, FDR-corrected) and patients with RTLE (*p* = 0.03, FDR-corrected).

## 4. Discussion

Cerebellar abnormality is a common phenotype of MTLE. The cerebellar abnormality contributed to cognitive impairment in MTLE (2, 3, 49). In this study, we applied group ICA and graph theoretical approaches to verbal fluency task-based functional MRI (fMRI) data. We divided the cerebellum into six functionally separated ICs and found disruption in functional connectivity between ICs in patients with RTLE. In LTLE, cerebellar centrality of the right lobule III was significantly increased compared to HC and patients with RTLE. These functional alterations were correlated with a decline in long-term memory in MTLE.

We separated the cerebellum into six functionally discrete components to identify cerebellar networks. Our cerebellar ICs and cerebral parcellations were similar to previously reported atlases. IC 3 and IC 6 contained cerebellar lobule IX, part of the DMN (50, 51), and were connected to cerebral DMN as expected. In our parcellation, IC 1 consists of the primary sensorimotor zone of the cerebellum, and IC 5 is the oculomotor network of the cerebellum (52). Similar to ICs generated with the MELODIC software (29) or the MICA toolbox (43), IC 2 and IC 6 in this study encompassed roughly symmetrical cerebellar regions (crus I and II) and were connected to the right and left FPN, respectively (53). Meanwhile, we noted some differences between our observations and those found in previous studies. In contrast to previous reports in the resting-state data, we noted greater activation in IC 6 than in IC 2 because the Chinese character fluency task was left-hemisphere dominant for right-handed participants. In addition, Alsady et al. observed a cerebellar IC connected to the cerebral DAN and the sensorimotor network. In this study, we separated the cerebellar oculomotor network IC 5, which was connected to the cerebral sensorimotor network, while cerebral DAN was connected to IC 4. Despite the minor discrepancy in group ICA, this study supported other studies regarding cerebellar segregation and cerebro-cerebellar connectivity.

During the task, functional connectivity between IC 3 and IC 5 was enhanced and functional connectivity from IC 6 to IC 4 was impaired in patients with RTLE compared to HCs and patients with LTLE. A mutual connection between IC 3 and IC 5 represented a bidirectional communication between DMN and the sensorimotor system in the cerebellum. Connectivity between the cerebral sensorimotor system and DMN was enhanced in drug-resistant epilepsy (54), frontal lobe epilepsy (55), juvenile myoclonic epilepsy (56), and generalized tonic–clonic seizures (57), and it was thought to lead to epileptic susceptibility (56, 57). Connectivity between the DMN and sensory regions caused lapses in certain ways (58). In addition, hypoconnectivity from IC 6 to IC 4 indicated disconnection between the left FPN, DMN, and DAN. In MTLE, connectivity between DAN and the executive control network was decreased in patients with cognitive impairment (59).

Degree centrality measures the number of suprathreshold connections linked to a certain node. In this study, the right lobule III in LTLE had significantly higher DC than that in RTLE and HCs. The cerebellum became a functional hub for the whole-brain network in MTLE. Garcia-Ramos et al. hypothesized that this is a compensatory reaction as the cerebellum was more integrated into the cerebral network and tried to compensate for the impaired cerebral function (28).

The Chinese version of the Information Test covered basic questions on geography, literature, history, and general knowledge. At the same educational level, the Information Test estimates long-term semantic memory (32). In RTLE, hyperconnectivity between IC 3 and IC 5 contributed to worse performance on the Information Test. In LTLE, higher compensatory DC of the right lobule III was correlated with higher Information Test scores. Though semantic memory was impaired at the same level for LTLE and RTLE in alphabetic languages (60, 61), our previous studies involving more participants reported worse Information Test scores in RTLE compared to HC and LTLE (32, 62), indicating that the Information Test might be a right-hemisphere dominant test in Chinese. Our results indicated that cerebellar disruption and compensation contributed to long-term memory in MTLE and were potential intervention targets for cognitive deficiency in MTLE.

The etiology of cerebellar alterations in TLE remains a controversial topic. Traditionally, cerebellar alterations were considered to be secondary due to seizures or disruption of cerebral networks. Studies focusing on anatomical damage of the cerebellum demonstrated a relationship between more severe cerebellar damage and a higher disease burden. In addition, the volume alteration of the vermis is correlated with the temporal lobe volume (63). However, functional studies of the cerebellum failed to confirm a relationship between disease burden and functional alterations (2, 3). Notably, cerebellar functional abnormalities also did not correlate with the clinical features in this study. Several points might explain the lack of correlation. First, a composite destructive and compensatory process might weaken the correlation between functional abnormalities and clinical features. Second, the cerebellar function was susceptible to ASM. The combinations and types of ASM could have biased the results.

Etiological and functional differences between LTLE and RTLE had been noted in previous studies. The language network (64) and the episodic memory network (65) were contralaterally shifted in LTLE but not in RTLE. Meanwhile, the fiber bundles of the alertness network were impaired, specifically in RTLE (66). The graph theoretical study also revealed different electrophysiological reorganizations in LTLE and RTLE, as functional connectivity was altered in the alpha band in LTLE and in the theta, beta, and gamma bands in RTLE (67). A multivariate pattern classification model constructed with cerebellar and cerebral structural connectivity achieved 93% accuracy in differentiating LTLE from RTLE (68). Consistent with our findings, Zanão et al. observed that the functional connectivity of DMN was enhanced in RTLE, compared to that in LTLE (69). Our result generalized previous findings to the cerebellum and strengthened the idea that LTLE and RTLE might be different epilepsy entities.

This study has limitations. First, ASM affects the results of fMRI (70). Correlation analysis demonstrated that cerebellar function was not biased by the number of ASMs. We also excluded patients who received phenytoin treatment, and we performed a sensitivity analysis excluding a patient who received topiramate. However, different types and combinations of ASM would still influence functional connectivity and correlation analysis. Second, the cerebellar alterations were explored only in fMRI data. The sample sizes of each patient group are also small. Nevertheless, we obtained detailed clinical information and applied robust methods, allowing us to detect cerebellar functional alterations under a stringent threshold. Correlation analysis also excluded potential clinical confounders that might bias the result. Future studies on the combination of structural and functional images in a larger cohort would provide further evidence of cerebellar involvement in MTLE.

## 5. Conclusion

We noted functional disruption and compensation in the cerebellum of patients with MTLE. Functional connectivity between cerebellar networks was modulated in RTLE, while the centrality of the right lobule III was increased in LTLE. These alterations were correlated with long-term memory in patients. Our results further support the cerebellar involvement in cognitive decline in MTLE and provided potential intervention targets for MTLE.

## Data availability statement

The raw data supporting the conclusions of this article will be made available by the authors, without undue reservation.

## Ethics statement

The studies involving human participants were reviewed and approved by the Ethics Committee of the Xiangya Hospital of Central South University. Written informed consent to participate in this study was provided by the participants' legal guardian/next of kin.

## Author contributions

YP, FX, LL, and BX contributed to the conception and design of the study. YP and KW undertook the analysis and drafted the manuscript. FX, KW, CL, LT, and XL organized the database. FX, KW, CL, LT, MZ, JH, YD, and GW collected the data. All authors contributed to the article and approved the submitted version.
